# Bis[(2-amino­phen­yl)methanol-κ^2^ *N*,*O*]bis­(nitrato-κ*O*)zinc(II)

**DOI:** 10.1107/S1600536810030485

**Published:** 2010-08-04

**Authors:** Majid Esmhosseini

**Affiliations:** a Department of Chemistry, University of Urmiyeh, Urmyieh, Iran

## Abstract

In the title compound, [Zn(NO_3_)_2_(C_7_H_9_NO)_2_], the Zn^II^ atom, lying on a twofold rotation axis, is six-coordinated in a distorted octa­hedral geometry by two N atoms and two O atoms from two (2-amino­phen­yl)methanol ligands and two O atoms from two monodentate nitrate anions. Inter­molecular N—H⋯O, O—H⋯O and C—H⋯O hydrogen bonds stabilize the crystal structure.

## Related literature

For related structures, see: Bandoli *et al.* (2002 ▶); Lewiński *et al.* (1998 ▶). 
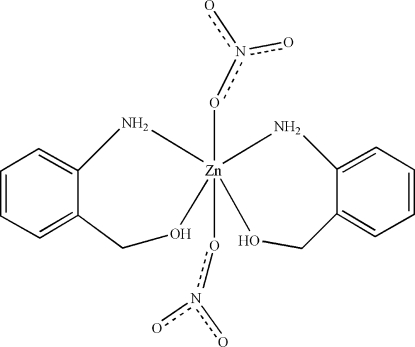


## Experimental

### 

#### Crystal data

[Zn(NO_3_)_2_(C_7_H_9_NO)_2_]
*M* *_r_* = 435.71
Orthorhombic, 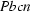

*a* = 23.386 (5) Å
*b* = 10.193 (2) Å
*c* = 7.3442 (15) Å
*V* = 1750.7 (6) Å^3^
*Z* = 4
Mo *K*α radiation
μ = 1.46 mm^−1^
*T* = 298 K
0.35 × 0.03 × 0.02 mm

#### Data collection

Bruker APEX CCD diffractometer
Absorption correction: multi-scan (*SADABS*; Sheldrick, 1996 ▶) *T*_min_ = 0.857, *T*_max_ = 0.980
14935 measured reflections
3005 independent reflections
2063 reflections with *I* > 2σ(*I*)
*R*_int_ = 0.140

#### Refinement

*R*[*F* ^2^ > 2σ(*F* ^2^)] = 0.088
*wR*(*F* ^2^) = 0.154
*S* = 1.26
3005 reflections
135 parameters
H atoms treated by a mixture of independent and constrained refinement
Δρ_max_ = 0.67 e Å^−3^
Δρ_min_ = −0.76 e Å^−3^

### 

Data collection: *SMART* (Bruker, 2007 ▶); cell refinement: *SAINT* (Bruker, 2007 ▶); data reduction: *SAINT*; program(s) used to solve structure: *SHELXTL* (Sheldrick, 2008 ▶); program(s) used to refine structure: *SHELXTL*; molecular graphics: *ORTEP-3* (Farrugia, 1997 ▶); software used to prepare material for publication: *WinGX* (Farrugia, 1999 ▶).

## Supplementary Material

Crystal structure: contains datablocks I, global. DOI: 10.1107/S1600536810030485/hy2332sup1.cif

Structure factors: contains datablocks I. DOI: 10.1107/S1600536810030485/hy2332Isup2.hkl

Additional supplementary materials:  crystallographic information; 3D view; checkCIF report

## Figures and Tables

**Table 1:** Selected bond lengths (Å)

Zn1—O1	2.142 (3)
Zn1—O2	2.190 (3)
Zn1—N1	2.108 (4)

**Table 2:** Hydrogen-bond geometry (Å, °)

*D*—H⋯*A*	*D*—H	H⋯*A*	*D*⋯*A*	*D*—H⋯*A*
N1—H1*C*⋯O3^i^	0.79 (6)	2.24 (6)	2.987 (5)	157 (6)
N1—H1*D*⋯O4^ii^	0.86 (7)	2.24 (7)	3.096 (5)	169 (7)
O1—H1*E*⋯O2^iii^	0.72 (7)	2.03 (7)	2.710 (4)	159 (7)
C1—H1*B*⋯O4^ii^	0.97	2.56	3.441 (6)	150

Symmetry codes: (i) 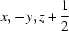; (ii) 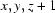; (iii) 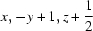.

**Fig. 1.**
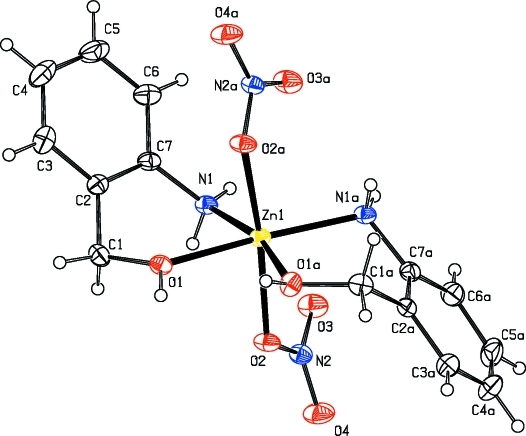
The molecular structure of the title compound. Displacement ellipsoids are drawn at the 30% probability level. [Symmetry code: (a) 1 - x, y, 1/2 - z.]

**Fig. 2.**
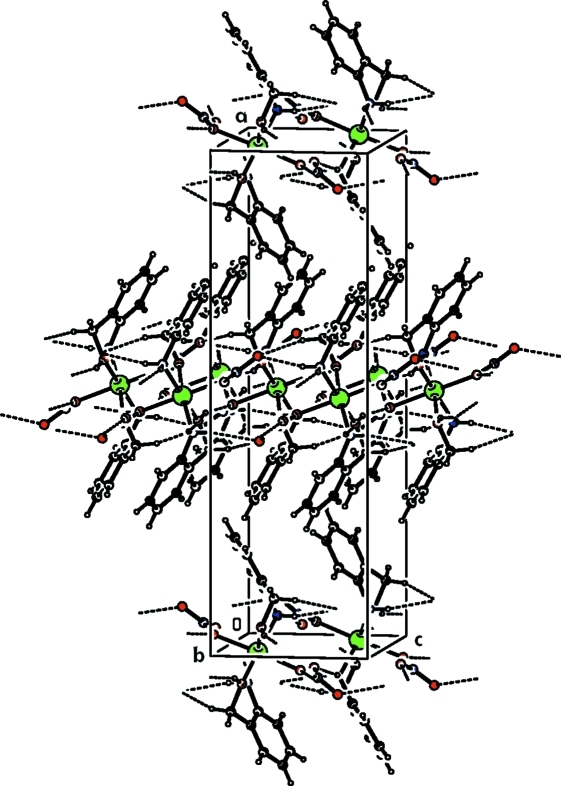
Crystal packing diagram for the title compound. Dashed lines denote hydrogen bonds.

## supplementary crystallographic information

### Comment

(2-Aminophenyl)methanol is a bidentate ligand. There is only two complexes with this ligand reported, such as those of Re (Bandoli *et al.*, 2002) and Al (Lewiński *et al.* 1998). We report herein the synthesis and structure of the title compound.

The asymmetric unit of the title compound (Fig. 1) contains half molecule. The Zn^II^ atom, lying on a twofold rotation axis, is six-coordinated in a distorted octahedral geometry by two N atoms and two O atoms from two (2-aminophenyl)methanol ligands and two O atoms from two nitrate anions (Table 1). Intermolecular N—H···O, O—H···O and C—H···O hydrogen bonds stabilize the crystal structure (Fig. 2, Table 2).

### Experimental

A solution of (2-aminophenyl)methanol (0.25 g, 2.00 mmol) in methanol (10 ml) was added to a solution of Zn(NO_3_)_2_.4H_2_O (0.26 g, 1.00 mmol) in methanol (10 ml) and the resulting colorless solution was stirred for 20 min at 313 K. This solution was left to evaporate slowly at room temperature. After one week, colorless needle crystals of the title compound were isolated (yield: 0.32 g, 73.4%).

### Refinement

C-bound H atoms were positioned geometrically and refined as riding atoms, with C—H = 0.93 (aromatic) and 0.97 (CH_2_) Å and *U*_iso_(H) = 1.2*U*_eq_(C). H atoms of the amino and hydroxy groups were located in a difference Fourier map and refined isotropically.

### Crystal data

**Table:**

[Zn(NO_3_)_2_(C_7_H_9_NO)_2_]	*F*(000) = 896
*M**_r_* = 435.71	*D*_x_ = 1.653 Mg m^−^^3^
Orthorhombic, *P**b**c**n*	Mo *K*α radiation, λ = 0.71073 Å
Hall symbol: -P 2n 2ab	Cell parameters from 1421 reflections
*a* = 23.386 (5) Å	θ = 2.2–32.0°
*b* = 10.193 (2) Å	µ = 1.46 mm^−^^1^
*c* = 7.3442 (15) Å	*T* = 298 K
*V* = 1750.7 (6) Å^3^	Needle, colorless
*Z* = 4	0.35 × 0.03 × 0.02 mm

### Data collection

**Table:**

Bruker APEX CCD diffractometer	3005 independent reflections
Radiation source: fine-focus sealed tube	2063 reflections with *I* > 2σ(*I*)
graphite	*R*_int_ = 0.140
φ and ω scans	θ_max_ = 32.0°, θ_min_ = 2.2°
Absorption correction: multi-scan (*SADABS*; Sheldrick, 1996)	*h* = −34→34
*T*_min_ = 0.857, *T*_max_ = 0.980	*k* = −15→13
14935 measured reflections	*l* = −9→10

### Refinement

**Table:**

Refinement on *F*^2^	Primary atom site location: structure-invariant direct methods
Least-squares matrix: full	Secondary atom site location: difference Fourier map
*R*[*F*^2^ > 2σ(*F*^2^)] = 0.088	Hydrogen site location: inferred from neighbouring sites
*wR*(*F*^2^) = 0.154	H atoms treated by a mixture of independent and constrained refinement
*S* = 1.26	*w* = 1/[σ^2^(*F*_o_^2^) + (0.0303*P*)^2^ + 3.223*P*] where *P* = (*F*_o_^2^ + 2*F*_c_^2^)/3
3005 reflections	(Δ/σ)_max_ = 0.004
135 parameters	Δρ_max_ = 0.67 e Å^−^^3^
0 restraints	Δρ_min_ = −0.76 e Å^−^^3^

### Fractional atomic coordinates and isotropic or equivalent isotropic displacement parameters (Å^2^)

**Table:**

	*x*	*y*	*z*	*U*_iso_*/*U*_eq_	
C1	0.3876 (2)	0.4320 (4)	0.3878 (6)	0.0324 (9)	
H1A	0.3663	0.5136	0.3834	0.039*	
H1B	0.3910	0.4056	0.5142	0.039*	
C2	0.35648 (17)	0.3286 (4)	0.2831 (5)	0.0270 (8)	
C3	0.3046 (2)	0.3574 (5)	0.1974 (7)	0.0408 (11)	
H3	0.2892	0.4413	0.2074	0.049*	
C4	0.2756 (2)	0.2623 (7)	0.0976 (8)	0.0519 (15)	
H4	0.2411	0.2826	0.0417	0.062*	
C5	0.2980 (2)	0.1394 (7)	0.0818 (8)	0.0499 (14)	
H5	0.2790	0.0768	0.0124	0.060*	
C6	0.3488 (2)	0.1067 (5)	0.1679 (6)	0.0381 (10)	
H6	0.3633	0.0220	0.1589	0.046*	
C7	0.37800 (17)	0.2015 (4)	0.2681 (5)	0.0279 (7)	
N1	0.43276 (16)	0.1728 (4)	0.3456 (5)	0.0262 (7)	
H1C	0.439 (2)	0.097 (6)	0.339 (8)	0.040 (15)*	
H1D	0.433 (3)	0.192 (7)	0.460 (9)	0.06 (2)*	
N2	0.44748 (15)	0.2148 (4)	−0.1135 (4)	0.0286 (7)	
O1	0.44352 (14)	0.4515 (3)	0.3116 (5)	0.0305 (7)	
H1E	0.457 (3)	0.510 (7)	0.344 (9)	0.054 (19)*	
O2	0.46612 (15)	0.3154 (3)	−0.0261 (4)	0.0316 (7)	
O3	0.46254 (17)	0.1044 (3)	−0.0629 (5)	0.0427 (8)	
O4	0.41587 (17)	0.2329 (4)	−0.2447 (5)	0.0484 (9)	
Zn1	0.5000	0.29214 (6)	0.2500	0.02484 (16)	

### Atomic displacement parameters (Å^2^)

**Table:**

	*U*^11^	*U*^22^	*U*^33^	*U*^12^	*U*^13^	*U*^23^
C1	0.038 (2)	0.030 (2)	0.029 (2)	0.0063 (18)	0.0021 (18)	−0.0027 (16)
C2	0.0273 (17)	0.0312 (19)	0.022 (2)	−0.0029 (15)	0.0007 (13)	0.0042 (13)
C3	0.031 (2)	0.047 (3)	0.045 (3)	0.003 (2)	0.0000 (18)	0.009 (2)
C4	0.030 (2)	0.074 (4)	0.052 (3)	−0.005 (2)	−0.012 (2)	0.006 (3)
C5	0.039 (3)	0.065 (4)	0.046 (3)	−0.021 (3)	−0.007 (2)	−0.005 (3)
C6	0.044 (3)	0.037 (2)	0.033 (2)	−0.010 (2)	−0.004 (2)	−0.001 (2)
C7	0.0324 (16)	0.0315 (17)	0.0199 (17)	−0.0051 (16)	−0.0010 (15)	0.0002 (17)
N1	0.0344 (18)	0.0186 (15)	0.0257 (17)	−0.0002 (14)	−0.0053 (14)	0.0003 (12)
N2	0.0364 (17)	0.0277 (16)	0.0218 (14)	−0.0003 (16)	−0.0019 (13)	−0.0022 (14)
O1	0.0335 (15)	0.0188 (13)	0.0391 (16)	−0.0032 (12)	−0.0028 (13)	−0.0081 (12)
O2	0.0494 (18)	0.0201 (14)	0.0253 (14)	−0.0007 (13)	−0.0107 (13)	0.0000 (11)
O3	0.061 (2)	0.0234 (15)	0.0440 (19)	0.0019 (15)	−0.0116 (17)	−0.0025 (14)
O4	0.062 (2)	0.053 (2)	0.0302 (15)	−0.0018 (17)	−0.0188 (18)	−0.0012 (19)
Zn1	0.0289 (3)	0.0202 (2)	0.0254 (3)	0.000	−0.0063 (3)	0.000

### Geometric parameters (Å, °)

**Table:**

C1—O1	1.436 (6)	C6—C7	1.394 (6)
C1—C2	1.494 (6)	C6—H6	0.9300
C1—H1A	0.9700	C7—N1	1.432 (5)
C1—H1B	0.9700	N1—H1C	0.79 (6)
C2—C7	1.394 (6)	N1—H1D	0.86 (7)
C2—C3	1.399 (6)	N2—O4	1.228 (5)
C3—C4	1.391 (8)	N2—O3	1.236 (5)
C3—H3	0.9300	N2—O2	1.286 (5)
C4—C5	1.363 (9)	Zn1—O1	2.142 (3)
C4—H4	0.9300	O1—H1E	0.72 (7)
C5—C6	1.386 (7)	Zn1—O2	2.190 (3)
C5—H5	0.9300	Zn1—N1	2.108 (4)
			
O1—C1—C2	109.9 (3)	Zn1—N1—H1C	114 (4)
O1—C1—H1A	109.7	C7—N1—H1D	111 (4)
C2—C1—H1A	109.7	Zn1—N1—H1D	100 (4)
O1—C1—H1B	109.7	H1C—N1—H1D	106 (6)
C2—C1—H1B	109.7	O4—N2—O3	123.0 (4)
H1A—C1—H1B	108.2	O4—N2—O2	118.4 (4)
C7—C2—C3	118.2 (4)	O3—N2—O2	118.6 (3)
C7—C2—C1	121.3 (4)	C1—O1—Zn1	122.6 (3)
C3—C2—C1	120.4 (4)	C1—O1—H1E	113 (5)
C4—C3—C2	120.8 (5)	Zn1—O1—H1E	115 (5)
C4—C3—H3	119.6	N2—O2—Zn1	119.9 (2)
C2—C3—H3	119.6	N1—Zn1—N1^i^	109.5 (2)
C5—C4—C3	120.0 (5)	N1—Zn1—O1	84.67 (14)
C5—C4—H4	120.0	N1^i^—Zn1—O1	165.41 (13)
C3—C4—H4	120.0	N1—Zn1—O1^i^	165.41 (13)
C4—C5—C6	120.7 (5)	N1^i^—Zn1—O1^i^	84.67 (14)
C4—C5—H5	119.6	O1—Zn1—O1^i^	81.37 (18)
C6—C5—H5	119.6	N1—Zn1—O2^i^	91.37 (13)
C5—C6—C7	119.6 (5)	N1^i^—Zn1—O2^i^	95.81 (13)
C5—C6—H6	120.2	O1—Zn1—O2^i^	86.87 (12)
C7—C6—H6	120.2	O1^i^—Zn1—O2^i^	83.69 (12)
C6—C7—C2	120.6 (4)	N1—Zn1—O2	95.81 (13)
C6—C7—N1	120.4 (4)	N1^i^—Zn1—O2	91.37 (13)
C2—C7—N1	118.8 (4)	O1—Zn1—O2	83.69 (12)
C7—N1—Zn1	114.6 (3)	O1^i^—Zn1—O2	86.88 (12)
C7—N1—H1C	110 (4)	O2^i^—Zn1—O2	167.55 (15)
			
O1—C1—C2—C7	59.5 (5)	O3—N2—O2—Zn1	−19.1 (5)
O1—C1—C2—C3	−120.7 (4)	C7—N1—Zn1—N1^i^	−126.8 (3)
C7—C2—C3—C4	−1.0 (7)	C7—N1—Zn1—O1	49.8 (3)
C1—C2—C3—C4	179.1 (5)	C7—N1—Zn1—O1^i^	66.7 (6)
C2—C3—C4—C5	−0.3 (8)	C7—N1—Zn1—O2^i^	136.5 (3)
C3—C4—C5—C6	1.6 (9)	C7—N1—Zn1—O2	−33.3 (3)
C4—C5—C6—C7	−1.6 (8)	C1—O1—Zn1—N1	0.8 (3)
C5—C6—C7—C2	0.3 (7)	C1—O1—Zn1—N1^i^	168.0 (5)
C5—C6—C7—N1	−174.9 (4)	C1—O1—Zn1—O1^i^	−175.0 (4)
C3—C2—C7—C6	1.0 (6)	C1—O1—Zn1—O2^i^	−90.9 (3)
C1—C2—C7—C6	−179.2 (4)	C1—O1—Zn1—O2	97.3 (3)
C3—C2—C7—N1	176.2 (4)	N2—O2—Zn1—N1	−46.2 (3)
C1—C2—C7—N1	−3.9 (6)	N2—O2—Zn1—N1^i^	63.6 (3)
C6—C7—N1—Zn1	118.9 (4)	N2—O2—Zn1—O1	−130.2 (3)
C2—C7—N1—Zn1	−56.4 (4)	N2—O2—Zn1—O1^i^	148.2 (3)
C2—C1—O1—Zn1	−48.3 (4)	N2—O2—Zn1—O2^i^	−171.1 (3)
O4—N2—O2—Zn1	161.5 (3)		

Symmetry codes: (i) −*x*+1, *y*, −*z*+1/2.

### Hydrogen-bond geometry (Å, °)

**Table:**

*D*—H···*A*	*D*—H	H···*A*	*D*···*A*	*D*—H···*A*
N1—H1C···O3^ii^	0.79 (6)	2.24 (6)	2.987 (5)	157 (6)
N1—H1D···O4^iii^	0.86 (7)	2.24 (7)	3.096 (5)	169 (7)
O1—H1E···O2^iv^	0.72 (7)	2.03 (7)	2.710 (4)	159 (7)
C1—H1B···O4^iii^	0.97	2.56	3.441 (6)	150

Symmetry codes: (ii) *x*, −*y*, *z*+1/2; (iii) *x*, *y*, *z*+1; (iv) *x*, −*y*+1, *z*+1/2.
